# Novel roles of non-coding brain RNAs in health and disease

**DOI:** 10.3389/fnmol.2014.00055

**Published:** 2014-06-26

**Authors:** Hermona Soreq

**Affiliations:** Laboratory of Molecular Neuroscience, Department of Biological Chemistry, The Edmond and Lily Safra Center of Brain Sciences, The Alexander Silberman Institute for Life Sciences, The Hebrew University of JerusalemJerusalem, Israel

**Keywords:** microRNAs, long non-coding RNAs, cholinergic signaling, schizophrenia, epilepsy, ischemic stroke, Alzheimer's disease, Parkinson's disease

Non-coding RNAs (ncRNAs), and in particular microRNAs (miRNAs) are rapidly becoming the focus of research interest in numerous basic and translational fields, and their importance for many aspects in brain functioning reveals novel roles and merits special discussion. The wide-scope, multi-targeted, and highly efficient manner of ncRNA regulatory activities draws attention to this topic by many, but the available research tools and experimental protocols are still insufficient, and their importance for many aspects in brain functioning keeps changing. Much of the research effort in this field has initially been devoted to cancer research, but the regulatory role of ncRNAs is considered global. Consequently, molecular neuroscientists picked it up as well, although the brain presents special challenges for ncRNA and miRNA research. To reflect the rapid recent development of ncRNA and miRNA research in the nervous system, this Research Topic eBook is focused on the search for and exploration of those ncRNAs and miRNAs whose activities modulate the multi-leveled functions of the eukaryotic brain in health and disease. It strives to cover the state of the art expertise and describe novel roles for known and recently identified ncRNAs and miRNAs and cover experimental approaches for identifying and establishing ncRNA-target relationships, reports of the affected pathways, inherited and acquired changes in ncRNA functioning and the use of ncRNA mimics and blockade tools for interference with their functions in health and disease.

This eBook covers several key topics of interest in the molecular neuroscience field that try to bridge the gap between ncRNAs, miRNAs, and the wider research community. As researchers, we are interested in advancing this field for the improvement of both basic and translational studies aimed at progressing toward better human health and wellbeing. Therefore, this volume is opened by a review contributed by the Gerhard Schratt group that presents a comprehensive characterization of the nuclear miRNA repertoire of post-mitotic neurons (Khudayberdiev et al., 2013). This is followed by a thorough discussion of the flexibility and stability of miRNAs in brain development and function that was written by the Christophe Beclin group (Follert et al., 2014) and by insights on the functional interactions between miRNAs and copy number variations in the aging brain contributed by the Ronald Bontrop group (Persengiev et al., 2013). Yet other authors focused their articles on particular neuronal roles of specific miRNAs. Thus, Alexander Murashov and Di Wu described the role of miRNA-431 in regulating axon regeneration in mature sensory neurons by targeting the Wnt antagonist Kremen1 (Wu and Murashov, 2013), while Bettina Nadorp presented a new view of the different genes involved in specific neurotransmission pathways as co-regulated by miRNAs (Nadorp and Soreq, 2014). To this end, she initiated a bioinformatics effort combined with *in vivo* experimental work to discover and validate the role of predicted overlapping microRNA regulators of acetylcholine packaging and degradation in neuroinflammation-related disorders.

Engineered animal models represent an important tool for exploring ncRNA and miRNA functions in the brain, and several of the articles in this eBook reflect this aspect. Some of the covered research efforts took global experimental approaches in diverse engineered animal models; thus, the Sebastian Kadener group reported Genome-wide assessment of post-transcriptional control in the fly brain, highlighting the rapid changes in this dynamic field of research (Mezan et al., 2013). Yet others referred to the diagnostic potential, like the Andre Fisher group that covered the rapidly evolving field of miRNA biomarkers for Central Nervous System disease (Rao et al., 2013). Hartl and Grunwald-Kadow and co-authors outlined new roles for “old” miRNAs in nervous system functions and diseases (Hartl and Grunwald Kadow, 2013). Another, even newer topic in this field is that of long ncRNAs in neurodevelopmental disorders, a subject which is likely to develop exponentially in the coming years and was the focus of an article by the Armaz Aschrafi group (van de Vondervoort et al., 2013).

The rapidly gained reputation of miRNAs lead to escalating numbers of joint basic-clinical studies, and many of those put a major emphasis on the nervous system diseases as related to changes in miRNAs. The most prevalent neurodegenerative disease, Alzheimer's disease was the focus of two separate articles: Sebastian Hebert and colleagues discussed the future prospects of circulating miRNAs to become a useful diagnostic tool and create novel biomarkers for early identification of Alzheimer's disease (Dorval et al., 2013), whereas the Euginia Wang group presented an in-depth study of the prospects of one specific miRNA to become such a biomarker (Bhatnagar et al., 2014): miRNA-34c, which was previously shown to associate with aging and whose levels are shown in our eBook to increase in the Alzheimer's circulating plasma. The next two articles shift the interest to nervous system diseases affecting younger patients, like chronic pain and epilepsy. Here, Michaela Kress and co-authors address the topics of pain regulation by miRNAs in nociceptive circuits as predictors of future clinical applications (Kress et al., 2013), and David Henshall covers the issue of miRNAs involvement in status epilepticus (Henshall, 2013). A key issue in miRNA research involves the emerging need to combine experimental work with state of the art biostatistics and bioinformatics analyses. Combined bioinformatics/genetics and miRNA studies appear in the Markus Nothen review of the highly focused role of miRNAs as the cause of schizophrenia in those rare patients who are 22q11.2 deletion carriers, and this study was expanded to discuss the possible implications for idiopathic disease at large (Forstner et al., 2013). MiRNAs in sensorineural diseases of the ear were the focus of a mini-review by the Karen Avraham group, and may be perceived as a first sign of new discoveries on miRNA contributions in sensory impairments (Ushakov et al., 2013). Ischemic stroke is another nervous system disease with an expanding impact in these days of continuously prolonged life expectancy in Western societies. In our eBook, Julie Anne Saugstad and co-workers discuss modified miRNAs following focal cerebral ischemia in male and female mouse brains (Lusardi et al., 2014).

Apart from the miRNAs themselves, our eBook also refers to the protein complexes involved in miRNA functioning, also in the context of neurodegenerative disease. The RISC complex and its causal involvement in Parkinson's disease is the focus of an article by the Matthew Wood group (Heman-Ackah et al., 2013). Last, but not least are expanded repeat diseases that were covered by two independent studies: RNA pathogenesis via Toll-like receptor-activated inflammation in expanded repeat neurodegenerative diseases by the Catherine Suter group (Richards et al., 2013) and Small ncRNAs as source of complexity added to the RNA pathogenic mechanisms in trinucleotide repeat expansion diseases (Marti and Estivill, 2013). ncRNAs are here to stay, and their impact on our brain's functioning at the physiology, cell biology, behavior, and immune levels is worth an in-depth journey.

## Conflict of interest statement

The author declares that the research was conducted in the absence of any commercial or financial relationships that could be construed as a potential conflict of interest.
